# Enhancing Wound Healing with Nanohydrogel-Entrapped Plant Extracts and Nanosilver: An In Vitro Investigation

**DOI:** 10.3390/molecules29215004

**Published:** 2024-10-22

**Authors:** Devadass Jessy Mercy, Anbazhagan Thirumalai, Saranya Udayakumar, Balasubramanian Deepika, Gopalarethinam Janani, Agnishwar Girigoswami, Koyeli Girigoswami

**Affiliations:** Medical Bionanotechnology, Faculty of Allied Health Sciences, Chettinad Hospital and Research Institute, Chettinad Academy of Research and Education, Chettinad Health City, Kelambakkam, Chennai 603103, India; jessy331mercy@gmail.com (D.J.M.); athiru1999@gmail.com (A.T.); usaranyaudayakumar@gmail.com (S.U.); deepikabalu70@gmail.com (B.D.); jananigopalarethinam98@gmail.com (G.J.)

**Keywords:** wound healing, natural products, nanotechnology, biocompatibility, scratch assay

## Abstract

Wound healing is a complex process that can be improved through advanced biomedical approaches. Incorporating nanopolymers and plant extracts into wound dressings offers a favorable strategy for promoting tissue repair. Nanopolymers provide a controlled environment for sustained drug release while also protecting the wound from external contaminants. When combined with bioactive compounds from plant extracts, which possess antioxidant, anti-inflammatory, and antimicrobial properties, this hybrid approach can accelerate healing, reduce infection, and improve tissue regeneration. Hence, in this study, we have synthesized alginate/gelatin hydrogel blended with only nanosilver (Alg/gel-Ag) and with nanosilver and plant extracts like aloe vera, curcumin, plantain peel extract, and *Calendula* flower petal extract (Alg/gel-AgP). The synthesized hydrogels were characterized using different photophysical tools, and the cytotoxicity effect was studied using a fibroblast cell line (V79). The antibacterial effect of the hydrogels was also observed against *E. coli* and *S. aureus*, determining the MIC and MBC. The wound healing in vitro was also assessed using scratch assay which depicted a rapid wound closure for Alg/gel-AgP compared to the untreated control and Alg/gel-Ag. The combined effect between nanotechnology and natural extracts represents a novel and effective approach for enhancing the wound healing process.

## 1. Introduction

Nanotechnology encompasses the exploration, formulation, and application of materials and devices characterized by structures at the nanometer scale, typically less than 100 nanometers in size. This discipline entails the manipulation and regulation of individual atoms and molecules to fabricate novel materials and devices exhibiting unique properties. Such advancements have a profound influence on the field of medical sciences, potentially facilitating innovative approaches for the diagnosis, treatment, and prevention of various diseases. In contemporary contexts, personalized medicine, including intelligent drug delivery systems and proteomic and genomic assessments, is gathering increased attention due to its capacity to customize therapeutic interventions to meet individual patient needs. Wound healing is the vital biological process that restores skin integrity after an injury. It progresses through several stages like blood clotting, infection, propagation, and restoration [1,2]. Basically, wounds are categorized into two different types: chronic wounds and acute wounds. A chronic wound is a type of wound where the wound is unable to heal within an expected time frame, whereas an acute wound is swiftly healed [3]. Diabetic ulcers and pressure sores are some of the types of chronic wounds [4]. Similarly, abrasions, cuts, and medical cuts are some of the types of acute wounds [3]. Wound healing constitutes a fundamental aspect of preserving the physiological integrity of the skin and the underlying tissues. The restoration of the wound site’s regular functionality encompasses various stages characterized by distinct activities [5]. Modern day wound healing materials enable the enhanced regulation of the healing environment and can accelerate the healing process; however, they may need increased financial investment, complexity, and specific limitations [6]. Traditional materials are typically more readily available and economically viable, yet they may not furnish the optimal conditions needed for effective wound healing, particularly in cases that involve intricate or chronic wounds [7].

Due to the presence of their versatile properties, nanopolymers have been utilized as a promising scaffold to entrap drugs for effective wound healing. Properties like biocompatibility, enhanced drug delivery, the inhibition of biofilm, and the rejuvenation of tissues impart a high level of effectiveness in these hydrogels. Compared with others, alginate/gelatin polymers offer a combination of all the above properties [8]. These polymers are derived from natural sources like seaweed and collagen, which naturally degrade without harming the tissues. The alginate/gelatin blended nanopolymers naturally deliver essential wound healing factors compared to other synthetic polymers. The factors, including suitable moisture in the wound environment, higher cell growth, increased tissue generation, drug delivery capability, and superior hemostatic properties, make them apt to be used in wound healing [8,9]. Similarly, the distinct properties of this blended nanopolymer make it a highly promising biopolymer for addressing the limitations of current wound dressings by enhancing the absorption of wound fluids, thus preventing bacterial infections and allergic reactions, leading to excellent wound healing performance [10]. The incorporation of nanofibers into alginate/gelatin composites resulted in hydrogels with improved stiffness and shorter gelation times, significantly enhancing their structural stability when subjected to stress [11]. In comparing alginate/hyaluronic acid (Alg-HA) and alginate/gelatin (Alg-Gel) hydrogels, the Alg-HA system exhibited lower cell proliferation, primarily due to a deficiency in cellular adhesion sites. The inclusion of gelatin in the alginate matrix significantly enhanced fibroblast proliferation, resulting in higher cell propagation rates in Alg-Gel hydrogels compared to untreated controls. This increase in cell attachment and proliferation was accompanied by improvements in swelling behavior, morphology, and mechanical properties. Although the Alg-HA combination exhibited a controlled and intricate pore structure, its mechanical properties showed considerable variability. At the same time, its swelling profile was comparable to that of the Alg and Alg-Gel systems [12].

Nanomaterials offer promising benefits in wound healing, comprising their antimicrobial properties, improved drug delivery mechanisms, and capacity to foster cellular interactions and tissue regeneration [13]. The nanomaterials incorporated with plant extracts in wound healing formulations yield numerous advantages due to their bioactive constituents, which provide extensive opportunities to enhance the healing process of wounds and sometimes contribute towards scar removal. This approach provides the natural healing properties of plant extracts while utilizing the advanced delivery and bioactivity enhancement capabilities of nanomaterials [14]. Phytochemical rich plant extracts possess diverse pharmacological properties for multi-targeted therapy that treat diseases better than single-compound pharmaceuticals. Silver is currently one of the most widely used topical antiseptic agents in wound dressings. Due to the increasing prevalence of infectious diseases and the growing challenge of antibiotic resistance, pharmaceutical companies and researchers are actively exploring novel antibacterial alternatives to address these issues effectively. Silver’s broad-spectrum antimicrobial activity makes it a valuable component in modern wound care strategies [15]. The silver nanoparticles synthesized from plant extracts reveal notable anti-inflammatory and antimicrobial effects, vital for preventing infections in chronic wounds [16]. The antimicrobial properties of silver have been recognized for decades, with research indicating that at low concentrations, it is safe for human application. Studies have demonstrated that reducing the particle size of silver nanoparticles (AgNPs) alters their electronic structure, thereby enhancing their antimicrobial efficacy. This improved activity is largely attributed to the slow release and oxidation of Ag+ ions in biological environments. Additionally, Ag+ ions can penetrate cellular membranes and interfere with cell division, ultimately leading to microbial death [17]. While silver exhibits potent antimicrobial properties, it can also modulate early inflammatory responses, which potentially delays the wound healing. It achieves this by regulating the production of the pro-inflammatory cytokine IL-6 and influencing neutrophil infiltration through the fibroblast growth factor signaling pathway. This dual action allows silver to not only combat infection but also suppress excessive inflammation, which may contribute to a delayed healing process [18]. Enhancing plant extracts with nanostructures increases their bioavailability and enables a controlled release at the wound site, promoting in-depth penetration into the skin layers [19]. A wound dressing material can also be stabilized by incorporating the plant extracts with electrospun nanofibers [20]. Similarly, bioactive compound-loaded nanoliposomes embedded in nanofibers significantly improve cell viability, thus promoting tissue regeneration [21].

*Calendula* is a highly versatile therapeutic agent, demonstrating efficacy against a range of conditions such as skin disorders, cancer, and gastrointestinal issues due to its rich content of bioactive compounds. These properties contribute to its extensive application in modern medicine. In addition to its health benefits, *Calendula* extracts are utilized in the pharmaceutical, food, and cosmetic industries, showcasing their multifunctionality. They are also employed as natural biomaterials in drug delivery systems, enhancing the effectiveness of treatments and offering broad therapeutic potential for various health conditions [22].

The wound-healing properties of aloe vera have been recognized since ancient times. The primary mechanism behind its healing effect is attributed to a mannose-rich polysaccharide, glucomannan, which interacts with gibberellin and growth factors to stimulate fibroblast propagation. This activation enhances collagen production in terms of quality and quantity, accelerating tissue repair. Recent research has demonstrated that aloe vera extract promotes the propagation of various cell lines and facilitates faster wound closure in both normal and diabetic models [23]. Additionally, its therapeutic use extends to alleviating mucocutaneous conditions, such as gingivitis [24], oral submucous fibrosis [25], and vaginal atrophy, as well as mitigating mucosal damage from chemotherapy and radiotherapy [26]. The topical application of aloe vera gel has also been shown to promote angiogenesis, increasing blood supply to the wound, thereby improving its metabolic needs and overall healing [23].

Plantain peel extract plays a significant role in wound healing due to its properties like controlled inflammation [27], decreased oxidative stress [28], and preventing infections, all of which are critical for faster healing. The extract also promotes collagen synthesis and enhances the proliferation of key cells like fibroblasts and keratinocytes, accelerating wound closure and improving tissue regeneration. Additionally, its ability to retain moisture fosters a suitable environment for healing and minimizes scarring [29].

Similarly, curcumin, a bioactive compound of turmeric, delivers major anti-tumor, anti-inflammatory, and antioxidant properties, enhancing the healing of wounds. It is derived from the root of turmeric. The ancient Chinese population used curcumin as a traditional medicine to improve blood circulation and reduce inflammation. The dietary supplements derived from curcumin are particularly popular on the market [30]. A nanocomposite scaffold was developed for bone repair by incorporating mesoporous nanoparticles into a polycaprolactone (PCL) and polyethylene glycol (PEG) matrix. The scaffolds enabled the controlled delivery of curcumin and rhBMP-2, with the optimized scaffold (7 wt% nanoparticles) showing improved mechanical strength, hydrophilicity, and a sustained release of active agents. These properties enhance scaffold biodegradation and cell viability, making the scaffold a promising candidate for bone tissue engineering and regenerative medicine applications [31]. Another study developed curcumin-loaded dendritic silica/titania nanoparticles (DSTNs) coated with polyethylenimine/folic acid (PEI-FA) for ultrasound-triggered release and combined chemo-sonodynamic therapy. The PEI-FA coating enhances cancer cell targeting and prevents premature drug release. Ultrasound activation generates reactive oxygen species, releasing curcumin into cancer cells. The system showed higher anticancer activity compared to curcumin or nanocarriers alone, making it a promising approach for targeted cancer therapy [32].

The plant extracts like plantain peel, aloe vera, *Calendula officinalis*, and curcumin solution contain bioactive compounds with potent antimicrobial, anti-inflammatory, and antioxidant properties, as identified by previous researchers, which prevent infections in wounds and their surroundings; these, in turn, lead to rapid healing by reducing infection and offering protection against the ROS activity. So, in this present study, we have synthesized a wound healing product composed of alginate/gelatin hydrogel blended with nanosilver (Alg/gel+Ag) and with nanosilver and plant extracts like plantain peel, aloe vera, *Calendula officinalis*, and curcumin solution (Alg/gel+AgP). In order to initiate rapid healing, nanosilver was combined with plant extracts and used as a conventional wound healing agent; we have taken the hydrogel containing nanosilver (Alg/gel+Ag) as a drug control throughout the study. The products were evaluated for their antimicrobial activity against microorganisms, and an in vitro wound healing assay was conducted after the complete physicochemical characterization of the products.

## 2. Results and Discussions

### 2.1. Phytochemical Composition Analysis

Various biochemical tests were performed to analyze the phytochemicals present in the plant extracts of plantain peel (Figure S2a), aloe vera (Figure S2b), and *Calendula officinalis* flower (Figure S2c). Table S1 shows the different phytochemical compositions present in plantain peel extract, aloe vera extract, and *Calendula officinalis* flower extract.

### 2.2. Plant Constituent Analysis

The secondary metabolites present in plant extracts such as plantain peel (Figure S2d), aloe vera (Figure S2e), and *Calendula officinalis* flower (Figure S2f) were analyzed using GC-MS and the molecules present are listed in Tables S2–S4, respectively. These secondary metabolites may contribute to effective wound healing.

### 2.3. Preparation of Plant Extracts and the Hydrogels

The plant extracts like plantain peel, aloe vera, *Calendula officinalis* flower, curcumin solution, and the hydrogels Alg/gel+Ag and Alg/gel+AgP were synthesized, rendering the process displayed in (Figure S1a–k).

### 2.4. Characterization Using Various Photophysical Tools

**Figure 1:**
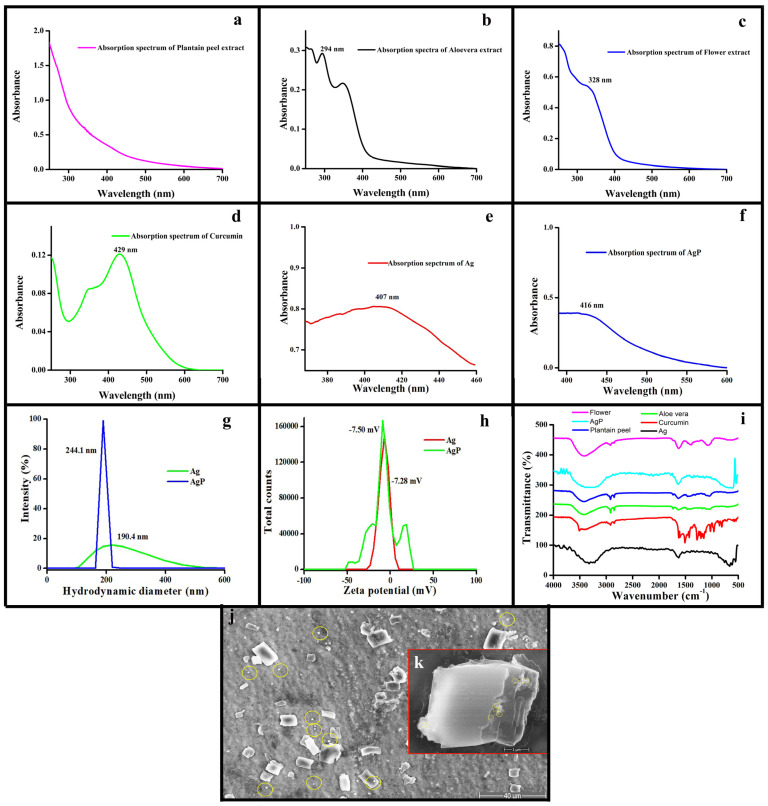
UV-visible spectra of plant extracts and hydrogels. (**a**) plantain peel extract; (**b**) aloe vera extract; (**c**) *Calendula officinalis* flower extract; (**d**) curcumin solution; (**e**) Alg/gel+Ag hydrogel; (**f**) Alg/gel+AgP hydrogel; (**g**) Hydrodynamic diameter of the Alg/gel+Ag and Alg/gel+AgP hydrogels; (**h**) zeta potential of the Alg/gel+Ag and Alg/gel+AgP hydrogels; (**i**) FTIR spectra of the Alg/gel+Ag and Alg/gel+AgP hydrogel. (**j**,**k**) SEM image of the Alg/gel+AgP hydrogel.

The synthesized hydrogels were characterized utilizing various techniques explained by Saranya et al. [33]. The UV-visible spectrophotometer was used to analyze the absorption spectra of the plant extracts and hydrogels Alg/gel+Ag and Alg/gel+AgP. A broad range between 250 and 450 nm was observed for plantain peel extract (Figure 1a), while the maximum absorption spectra were recorded at 294 nm for aloe vera extract (Figure 1b), 328 nm for flower extract (Figure 1c), 429 nm for curcumin solution (Figure 1d), 409 nm for hydrogel Alg/gel+Ag (Figure 1e), and 416 nm for hydrogel Alg/gel+AgP (Figure 1f), respectively. The maximum absorption spectra of the hydrogel Alg/gel+Ag and Alg/gel+AgP in the 400–420 nm range indicate the presence of AgNps blended in the hydrogel. The hydrodynamic diameter of the hydrogel Alg/gel+Ag was 190.4 nm, and when the plant extracts were incorporated alongside AgNps, the hydrodynamic diameter increased to 244.1 nm (Figure 1g). This increase in size indicates that the plant extracts likely interacted with the hydrogel matrix and AgNps, resulting in a more complex and expanded structure. Such interaction could enhance the overall stability and bioactivity of the hydrogel system. The zeta potential value for hydrogel Alg/gel+Ag was found to be −7.28 mV, and for hydrogel Alg/gel+AgP, it was found to be −7.50 mV (Figure 1h), showing that the hydrogel Alg/gel+AgP was moderately stable and interacted with the surface of hydrogel matrix or AgNps, slightly enhancing the surface charge compared to the hydrogel Alg/gel+Ag. FTIR was used to identify the characteristic bonds present in hydrogels Alg/gel+Ag and Alg/gel+AgP (Figure 1i). Tables S5 and S6 explain the conformational chemical bonds and functional groups of the prepared plant extracts and the hydrogels Alg/gel+Ag and Alg/gel+AgP. The chemical profiles of various plant extracts, including plantain peel, aloe vera, *Calendula officinalis*, and curcumin, showcase a diverse range of functional groups and conformational chemical bonds that contribute to their therapeutic effects. Plantain peel extract is characterized by hydroxyl and carbonyl groups alongside aliphatic and aromatic structures, enhancing its antioxidant and antimicrobial properties [34]. Aloe vera predominantly features hydroxyl groups in its polysaccharides, facilitating moisture retention and wound healing, while its anthraquinones impart analgesic and anti-inflammatory benefits [35,36]. *Calendula officinalis* extract contains similar hydroxyl groups and double bonds, contributing to their anti-inflammatory and antioxidant activities [37]. Curcumin, the primary active compound in turmeric, is distinguished by its hydroxyl functional groups within an aromatic framework, supporting its antioxidant and bioactive properties [38]. The hydrogel Alg/gel+Ag was characterized by the presence of the hydroxyl group and the amine group, which contribute to its gelation properties and biocompatibility. The hydrogel Alg/gel+Ag was characterized by the presence of nitro compounds, phenols, the hydroxyl group, and the amine group, which also contribute to its gelation properties and biocompatibility. Additionally, conformational chemical bonds, including ionic bonds formed with cations, hydrogen bonds, and potential covalent bonds during cross-linking, enhance its structural integrity and stability.

A Hi-Resolution Scanning Electron Microscope (Thermo Scientific, Waltham, MA, USA) was used to analyze the morphological characteristics of the synthesized hydrogel Alg/gel+AgP. (Figure 1j) shows the blended nanosilver with alginate/gelatin hydrogel. The presence of nanosilver was confirmed by the sphere shape particle with an average size of 180 nm (Figure 1k).

### 2.5. Cell Toxicity Evaluation

**Figure 2:**
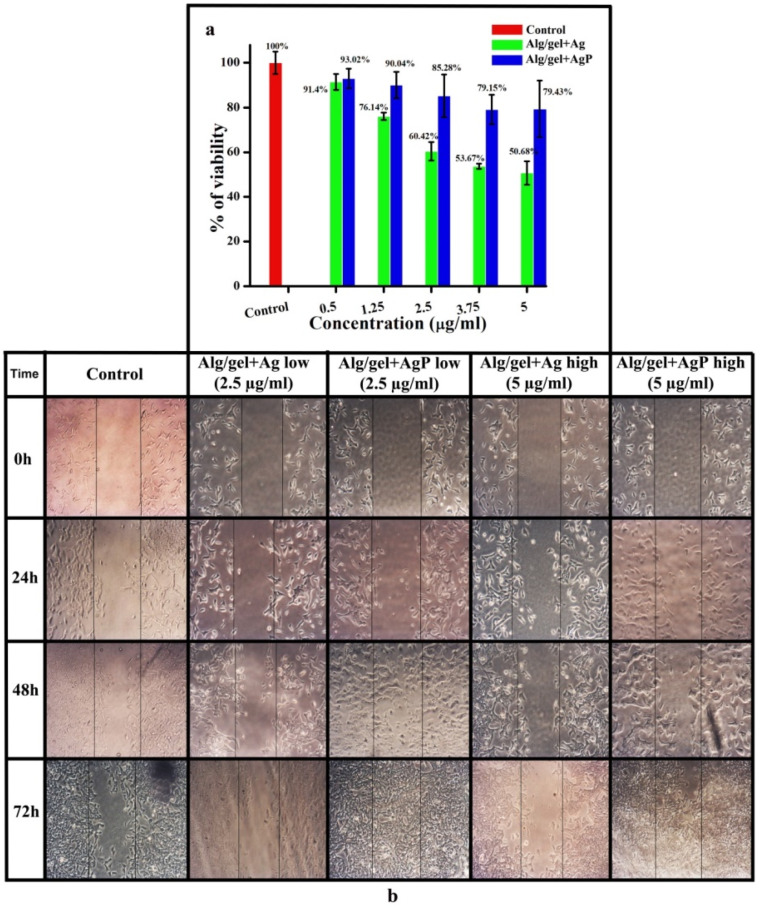
(**a**) In vitro analysis of the synthesized hydrogels Alg/gel+Ag and Alg/gel+AgP using MTT assay; (**b**) wound healing assay performed on V79 cell line with the hydrogels Alg/gel+Ag and Alg/gel+AgP.

The in vitro cytotoxicity of the hydrogels Alg/gel+Ag and Alg/gel+AgP in V79 cells was determined using MTT assay. From the graph (Figure 2a), we observed that hydrogel Alg/gel+AgP at its highest concentration (5 µg/mL) had less effect in killing the fibroblast cells compared to the hydrogel Alg/gel+Ag. This shows that the hydrogel Alg/gel+AgP has increased the specificity and efficiency of the extract. The cell viability percentage of the hydrogels Alg/gel+AgP (5 µg/mL) and Alg/gel+Ag (5 µg/mL) was found to be 79 ± 12% and 50 ± 5%, respectively.

### 2.6. Wound Healing Assay

The scratch was created in V79 cells, and once every day, it was treated with 2.5 µg/mL and 5 µg/mL of the hydrogels Alg/gel+Ag and Alg/gel+AgP for a period of 72 h (Figure 2b). The scratch closure was captured using an inverted microscope at 40× magnification. The figure implies a 98% scratch closure in Alg/gel+AgP hydrogel-treated cells, whereas it was 56% in Alg/gel+Ag hydrogel-treated cells and 67% in control cells after 72 h. The scratch was not closed even after 72 h in Alg/gel+Ag and control. Hence, when compared with the hydrogel Alg/gel+Ag, the hydrogel Alg/gel+AgP promotes enhanced cell migration and proliferation. This indicates that the hydrogel Alg/gel+AgP is highly effective and more specific than the hydrogel Alg/gel+Ag in promoting the regeneration of tissue, which leads to rapid wound healing.

### 2.7. Cell Viability Assay

**Figure 3:**
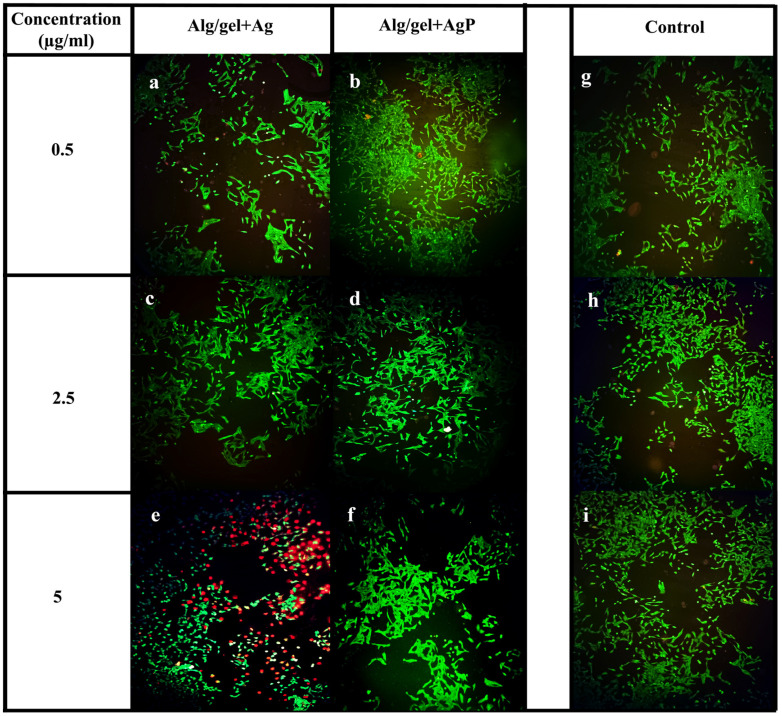
Cell viability assay performed on V79 cell line with the synthesized hydrogels Alg/gel+Ag and Alg/gel+AgP. (**a**,**c**,**e**) the hydrogel Alg/gel+Ag treated cells with the concentration of 0.5 µg/mL, 2.5 µg/mL and 5 µg/mL. (**b**,**d**,**f**) the hydrogel Alg/gel+AgP treated cells with the concentration of 0.5 µg/mL, 2.5 µg/mL and 5 µg/mL, respectively. (**g**,**h**,**i**) the untreated cells (control).

The viability of the cells treated with the hydrogels Alg/gel+Ag and Alg/gel+AgP was determined and visualized using a fluorescence microscope (Figure 3). The green fluorescence indicates the live cells, and the red fluorescence indicates the dead cells. In the untreated control group (Figure 3g–i) and the hydrogel Alg/gel+AgP (Figure 3b,d,f)-treated groups across all concentrations, 98% of live cells were consistently observed. In contrast, the hydrogel Alg/gel+Ag-treated group at the highest concentration showed a significant increase in dead cells of 93% (Figure 3e), along with notable morphological changes. These findings indicate that the hydrogel Alg/gel+Ag exhibited high toxicity at the highest concentration but was not toxic at the lower concentrations (Figure 3a,c). On the other hand, the hydrogel Alg/gel+AgP demonstrated no toxicity at any of the tested concentrations. This suggested that the synthesized hydrogel Alg/gel+AgP has the potential to promote wound healing without inducing cytotoxicity. Similarly, Aldagi et al. studied the effects of polymers like carboxymethyl cellulose, alginate, and gelatin hydrogel containing doxycycline (CMC/Alg/Gel/1% *w*/*v* DOX hydrogels) for the healing of pressure ulcers. A total of 56.66% of wound closure was observed in the CMC/Alg/Gel/1% *w*/*v* DOX hydrogels-treated group [39].

### 2.8. Analysis of Bactericidal Effect

**Figure 4:**
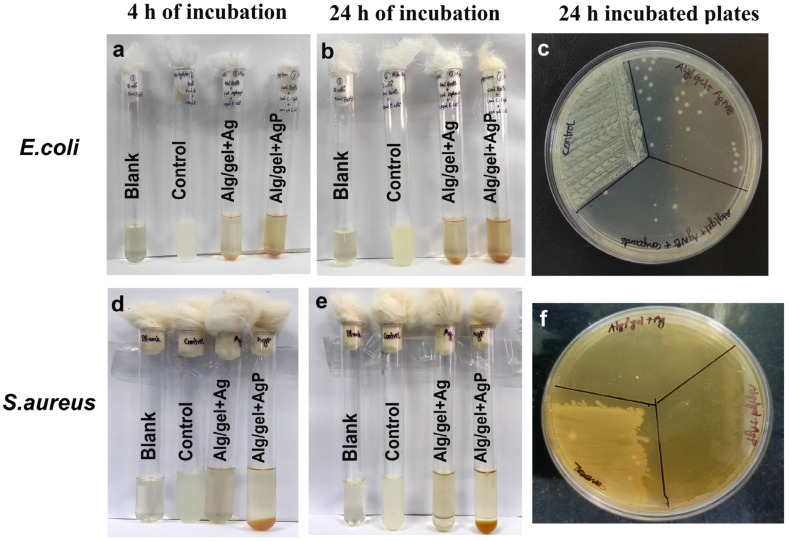
Assessment of antibacterial activity of the synthesized hydrogels Alg/gel+Ag and Alg/gel+AgP against *E. coli* and *S. aureus*: (**a**) 4 h incubated tubes treated with both the hydrogels studied against *E. coli*; (**b**) 24 h incubated tubes treated with both the hydrogels studied against *E. coli*; (**c**) 24 h incubated plates of the above culture tubes, against *E. coli*; (**d**) 4 h incubated tubes treated with both the hydrogels studied against *S. aureus*; (**e**) 24 h incubated tubes treated with both the hydrogels against studied *S. aureus*; (**f**) 24 h incubated plates of the above culture tubes against *S. aureus.*

The antibacterial activity of the hydrogels Alg/gel+Ag and Alg/gel+AgP was studied against *E. coli* and *S. aureus* (Figure 4). The incubated tubes treated with hydrogels showed less turbidity when compared to negative control, which indicates the bactericidal effect of the hydrogels Alg/gel+Ag and Alg/gel+AgP (Figure 4a,b,d,e). The above mentioned cultures were plated and showed less colony formation in *E. coli* treated with hydrogel Alg/gel+AgP when compared to hydrogel Alg/gel+Ag (Figure 4c). However, in *S. aureus*, there was merely a single colony formed in both Alg/gel+Ag and Alg/gel+AgP hydrogel (Figure 4f). This indicates that the Alg/gel+AgP showed a prominent effect on *S. aureus* when compared to *E. coli*, and in *E. coli*, the killing effect was higher for the hydrogel containing the plant extracts. Similarly, a multifunctional wound healing material was prepared and loaded with chamomile extract and silver sulfadiazine. This inhibited the synergistic effect on bacterial growth, thus protecting from the wound site [40].

### 2.9. Determination of MIC Value

**Figure 5:**
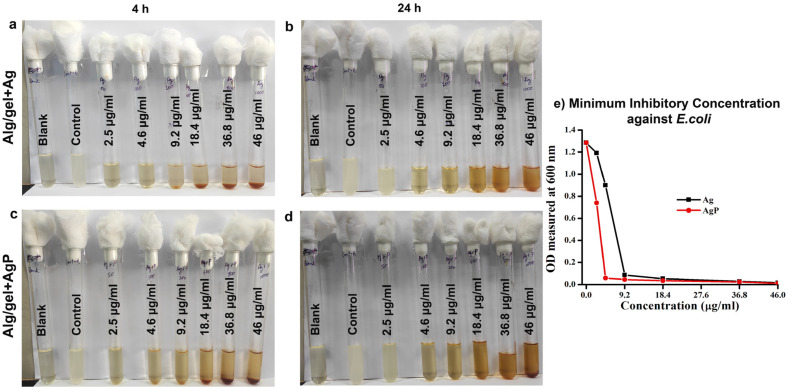
MIC of the synthesized hydrogels Alg/gel+Ag and Alg/gel+AgP against *E. coli:* (**a**) 4 h incubated tubes treated with the hydrogel Alg/gel+Ag; (**b**) 24 h incubated tubes treated with the hydrogel Alg/gel+Ag; (**c**) 4 h incubated tubes treated with the hydrogel Alg/gel+AgP; (**d**) 24 h incubated tubes treated with the hydrogel Alg/gel+AgP; (**e**) graph illustrating the MIC of the hydrogels Alg/gel+Ag and Alg/gel+AgP against *E. coli.*

**Figure 6:**
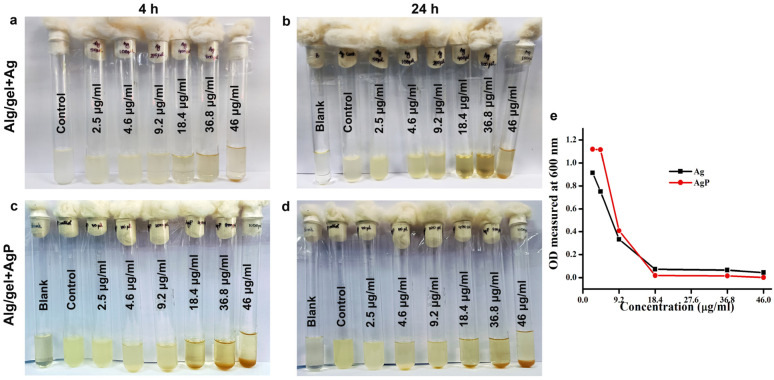
MIC of the synthesized hydrogels Alg/gel+Ag and Alg/gel+AgP against *S. aureus;* (**a**) 4 h incubated tubes treated with the hydrogel Alg/gel+Ag; (**b**) 24 h incubated tubes treated with the hydrogel Alg/gel+Ag; (**c**) 4 h incubated tubes treated with the hydrogel Alg/gel+AgP; (**d**) 24 h incubated tubes treated with the hydrogel Alg/gel+AgP; (**e**) graph illustrating the MIC of the hydrogels Alg/gel+Ag and Alg/gel+AgP against *S. aureus.*

Following the bactericidal activity, the MIC of the Alg/gel+Ag hydrogel and Alg/gel+AgP (Figure 5 and Figure 6) was also determined against *E. coli* and *S. aureus*. The MIC of the *E. coli* treated with the hydrogel Alg/gel+Ag and the hydrogel Alg/gel+AgP was found to be 18.4 µg/mL and 9.2 µg/mL, respectively (Figure 5a,b), and a corresponding graph was plotted to illustrate the result (Figure 5e). This indicates that the hydrogel Alg/gel+AgP possesses a greater inhibition of bacteria even in lower concentrations compared to hydrogel Alg/gel+Ag. Similarly, the MIC of the *S. aureus* treated with the hydrogel Alg/gel+Ag and the hydrogel Alg/gel+AgP was found to be 18.4 µg/mL (Figure 6c,d). Though both hydrogels have the same concentration, the hydrogel Alg/gel+AgP exhibited significant bacterial inhibition, which was plotted as a graph (Figure 6e).

## 3. Materials and Methods

### 3.1. Materials

Sodium alginate, LB broth, nutrient agar, Antibiotic/Antimycotic solution, MTT (3-(4,5-Dimethlythiazol-2-yl)-2,5-Diphenyltetrazolium Bromide), and Dulbecco’s Modified Eagle Medium solution were purchased from HiMedia, Maharashtra, India. Acetone, silver nitrate, glutaraldehyde, and curcumin were purchased from SRL. Calcium chloride and dimethyl sulfoxide were purchased from RANKEM (Bhiwandi City, India). Gelatin (Bloom 175) and Fetal Bovine Serum were purchased from SIGMA and Gibco, (Waltham, MA, USA), respectively. The V79 cell line was purchased from NCCS, Pune (Pune, India). The plantain was bought from a local market at Sembakkam, and aloe vera was obtained from Chettinad Organics (Tamil Nadu, India). The plant authentication was obtained from Siddha Central Research Institute, Ministry of AYUSH, Govt. of India. The *Calendula officinalis* flower powder was purchased online. The molecular formulae and the purity levels of all chemicals used in the study are given in Table S7.

### 3.2. Preparation of Plantain Peel Extract (Musa × paradisiaca Linn)

The *Musa × paradisiaca Linn* extract was prepared by following and slightly modifying the methods of Daverey et al. and Sofini et al. [41,42]. The plantain was procured, and the peels were collected, completely washed, and diced into pieces. These were then shade dried (Figure S1c) for about a week and oven dried for 30 min at 60 °C. After drying, a pestle and mortar were utilized to grind the plantain peel to acquire a finely ground powder. The plantain peel extract was prepared by weighing 3 g of the ground powder and dissolving it in 120 mL of distilled water. Further, the mixture was stirred thoroughly for 45 min utilizing a magnetic stirrer and then filtered through a Whatman filter paper. The obtained plantain peel extract was stored at 4 °C for future use (Figure S1d).

### 3.3. Preparation of Aloe Vera Extract (Aloe barbadensis Miller)

The aloe vera extract was prepared following the method described by Sofini et al. [42]. Aloe vera leaves were acquired and thoroughly washed, after which the outer peel was removed, and the pulp was diced into pieces. Approximately 3 g of the pulp was mixed with 120 mL of distilled water and continuously stirred and heated for about 6 h at 40 °C. After this period, the solution was filtered using a muslin cloth and stored at 4 °C (Figure S1a).

### 3.4. Preparation of Flower Extract (Calendula officinalis)

The *Calendula officinalis* flower extract was prepared using the method described by Olfati et al. [43] with minor modifications. The *Calendula officinalis* flower powder was commercially purchased and used (Figure S1e). A total of 3 g of the flower powder was weighed and dissolved in 120 mL of distilled water and boiled for 4–5 min at 100 °C. The boiled mixture was allowed to cool down at room temperature and then filtered using Whatman filter paper. The filtered flower extract was further stored at (Figure S1f) 4 °C.

### 3.5. Preparation of Curcumin Solution

The curcumin solution was prepared based on the method by Sofini et al. [42], with minor adjustments. A total of 0.25 g of curcumin was weighed and dissolved completely with 500 µL of dimethyl sulfoxide (DMSO). It was then diluted with 100 mL of distilled water and sonicated for 10 min to obtain the curcumin solution (Figure S1b).

### 3.6. Analysis of the Phytochemical Composition of the Prepared Plant Extracts

The phytochemical composition of the prepared plant extracts was analyzed qualitatively, following the methods outlined previously [44,45].

### 3.7. Analysis of the Plant Constituents Present in the Prepared Plant Extracts

Gas chromatography-mass spectroscopy (GC-MS) (QP2010 Shimadzu, Kyoto, Japan) was utilized to analyze the plant constituents present in the prepared plant extracts. A 1 μL aliquot of each plant extract was individually diluted with methanol and introduced into a column. The column temperature was initially set at 60 °C and then ramped from 60 °C to 320 °C over a period of 3 min. The injection port was maintained at 260 °C. The plant extracts were manually injected in split mode with a 5:1 ratio, and helium served as the carrier gas at a flow rate of 1.25 mL/min. The total elution time for the sample was 45 min. Compound identification was based on retention time, fragmentation patterns, and comparison with the NIST mass spectral database using automated mass spectral deconvolution and identification software.

### 3.8. Synthesis of Alginate/Gelatin Hydrogel Blended with Only Nanosilver (Alg/gel+Ag)

The alginate/gelatin hydrogel Alg/gel+Ag was synthesized following the methods explained by Baukum et al., with slight modifications [46]; 0.4 g of the gelatin was weighed and dissolved in 10 mL distilled water in a beaker with a mild heat of 50 °C and stirred until complete dissolution. After dissolving, 10 mL of acetone was added quickly, stirred for 8 min, and turned off. The sediment was allowed to settle down, and the supernatant was discarded completely. To the sediment, 7 mL of distilled water was added, and the stirring was turned on and continued for 10 min with a mild heat of 50 °C. Then, 1 g of 2% sodium alginate was weighed and dissolved in 50 mL distilled water under stirring with a mild heat of 40 °C to obtain the alginate solution. About 2 mL of the prepared alginate solution was added to the beaker containing the dissolved gelatin nanoparticle and stirred continuously for 10 min with a mild heat at 50 °C to obtain a homogenous solution. After obtaining the homogenous solution, 320 µL of 1% silver nitrate solution was added and stirred for 3 min without heat. Further, the solution was irradiated with UV for 1 h 30 min with continuous stirring. After 1 h 30 min of irradiation and stirring, 250 µL of 1% glutaraldehyde solution was added and stirred for 10 min. To this solution, about 3.1 mL of distilled water was added, and finally 60 µL of calcium chloride was added and stirred for 30 min. The final Alg/gel+Ag hydrogel was prepared after 30 min of stirring. The Alg/gel+Ag hydrogel was stored at 4 °C until use.

### 3.9. Synthesis of Alginate/Gelatin Blended Hydrogel with Nanosilver and Plant Extracts (Alg/gel+AgP)

The alginate/gelatin blended hydrogel Alg/gel+AgP was synthesized following the methods explained by Baukum et al., with slight modifications [46]; 0.4 g of the gelatin was weighed and dissolved in 10 mL distilled water in a beaker with a mild heat of 50 °C, stirring until complete solubilization. After dissolving, 10 mL of acetone was added quickly, stirred for 8 min, and turned off. The sediment was allowed to settle down, and the supernatant was discarded completely. To the sediment, 7 mL of distilled water was added, and the stirring was turned on and continued for 10 min with a mild heat of 50 °C. Then, 1 g of 2% sodium alginate was weighed and dissolved in 50 mL distilled water under stirring with a mild heat of 40 °C to obtain the alginate solution. About 2 mL of the prepared alginate solution was added to the beaker containing the dissolved gelatin nanoparticle and stirred continuously for 10 min with mild heat at 50 °C to obtain a homogenous solution. After obtaining the homogenous solution, 320 µL of 1% silver nitrate solution was added and stirred for 3 min without heat. Further, the solution was irradiated with UV for 1 h 30 min with continuous stirring. After 1 h 30 min of irradiation and stirring, 250 µL of 1% glutaraldehyde solution was added and stirred for 10 min. Then, 3.1 mL of plant extracts (1 mL of plantain peel extract, 1 mL of aloe vera extract, 1 mL of *Calendula officinalis* flower extract, and 100 µL of curcumin solution) was added and stirred for 10 min. The final concentrations of the extracts were 2.5% for plantain peel, aloe vera, *Calendula officinalis* flower, and 0.025% for curcumin. Finally, 60 µL of calcium chloride was added and stirred for 30 min. The final Alg/gel+AgP hydrogel was prepared after 30 min of stirring. The Alg/gel+AgP hydrogel was stored at 4 °C until use.

### 3.10. Characterization of the Synthesized Hydrogels Alg/gel+Ag and Alg/gel+AgP

The prepared plant extracts and synthesized hydrogels Alg/gel+Ag and Alg/gel+AgP were characterized based on the methods used in our previous studies. A Shimadzu UV-1800 spectrophotometer was utilized to analyze optical absorption. A Malvern ZS90 (Worcestershire, UK) particle size analyzer instrument was used to measure the stability and size while a Bruker ALPHA FTIR/ATR spectrometer was used to analyze the functional groups present in the prepared plant extracts like plantain peel, aloe vera, *Calendula officinalis* flower, curcumin solution, and the synthesized hydrogels Alg/gel+Ag and Alg/gel+AgP. A Thermo Scientific Apreo S Hi-Resolution Scanning Electron Microscope (HRSEM) was utilized to analyze the morphological characteristics of the hydrogel Alg/gel+AgP.

### 3.11. Cytotoxic Assessment

The in vitro cytotoxic assessments were carried out by following Pemula et al. and Saranya et al. with minor modifications [33,47]. MTT assay was conducted to assess the cytotoxicity of the synthesized hydrogels Alg/gel+Ag and Alg/gel+AgP in normal fibroblasts. Chinese Hamster lung fibroblast cells (V79 cells) were procured from NCCS Pune. The cells were cultured in 48-well plates with a seeding density of approximately 10^4^ cells/well. The cells were maintained in the exponential phase and cultured in Dulbecco’s Modified Eagle Medium (DMEM), added with 10% Fetal Bovine Serum (FBS) and 1% antibiotic/antimycotic solution. CO_2_ incubator (a sterile incubator that contains 5% CO_2_ and a humidified atmosphere with a temperature of 37 °C) was used to keep the cells. The cells were treated with different concentrations of hydrogel Alg/gel+Ag (0.5 µg/mL, 1.25 µg/mL, 2.5 µg/mL, 3.75 µg/mL, 5 µg/mL) and hydrogel Alg/gel+AgP (0.5 µg/mL, 1.25 µg/mL, 2.5 µg/mL, 3.75 µg/mL, 5 µg/mL), respectively, and untreated cells were used as a drug control. Following treatment, MTT was added to the cells incubated for 4 h in a CO_2_ incubator. Post-incubation, the purple formazan crystals were dissolved using DMSO, and the optical density was measured at 570 nm to calculate the percentage of viable cells. The percentage of viable cells was calculated as follows:

$$\begin{array}{c} \mathrm{Cell}\mathrm{viability}(\%)=\\ \frac{O.D.\mathrm{at}570\mathrm{nm}\mathrm{of}\mathrm{the}\mathrm{treated}\mathrm{cells}}{O.D.\mathrm{at}570\mathrm{nm}\mathrm{of}\mathrm{the}\mathrm{untreated}\mathrm{control}\mathrm{cells}}\;\times \;100 \end{array}$$

The experiment was conducted in triplicate.

### 3.12. In Vitro Cell Migration Assay

Any wound healing material will have a rapid cell migration and proliferation properties. The migration of the cells was studied using cell migration or scratch assay following the method of Shurfa et al. with minor modifications [48]. V79 cells were inoculated on a 35 mm Petri dish and cultured in complete DMEM as previously detailed, subsequently being incubated in a CO_2_ incubator. Upon achieving 80% confluency, a linear scratch was effectuated utilizing a sterile 100 µL pipette tip. The detached cells were gently rinsed and subsequently replenished with fresh medium. The cells were then subjected to treatment with (2.5 µg/mL, 5 µg/mL) concentrations of hydrogel Alg/gel+Ag and (2.5 µg/mL, 5 µg/mL) concentrations of hydrogel Alg/gel+AgP, followed by an incubation period of 24 h. The dosages were established based on the outcomes of the MTT assay. Untreated cells served as the control group. Microscopic images of the scratch were captured using an inverted microscope (Olympus CKX53) at a magnification of 40 X. Images were obtained immediately post-scratch and at 24 h, 48 h, and 72 h to quantify the areas throughout the treatment duration. The acquired images were subsequently analyzed employing Magnus (MagVision, 3.7) software. The proportion of the area that remained unclosed was calculated as follows:

$$\mathrm{Percentage}\mathrm{of}\mathrm{area}\mathrm{unclosed}=\frac{\mathrm{Final}\mathrm{unclosed}\mathrm{area}-\mathrm{Initial}\mathrm{unclosed}\mathrm{area}}{\mathrm{Initial}\mathrm{unclosed}\mathrm{area}}\;\times \;100$$

### 3.13. Live and Dead Cell Assay

Further verification of the in vitro biocompatibility of our products (Alg/gel+Ag and Alg/gel+AgP) was conducted by live dead cell assay after treatment with the hydrogels at different concentrations. The live dead assay was performed using the method explained by Karthick et al. [49]. The assay was carried out on Chinese Hamster lung fibroblast cells (V79 cells) which were obtained from NCCS Pune. The V79 cells were grown on the sterilized cover slip placed inside 35 mm Petri plates along with DMEM, 1% antibiotic solution, and 10% FBS. The plates were incubated in a CO_2_ incubator for 24 h and treated the next day by providing both the hydrogels with the concentrations of 0.25 µg/mL, 2.5 µg/mL, and 5 µg/mL, respectively. The stock concentration of 1.2 mM Acridine orange (AO) and 1.9 mM Ethidium bromide (EtBr) was newly prepared. In 2 mL PBS solution, 2 µL EtBr and 5 µL AO were added and used as a dye to stain the cover slip loaded with the cells. This process was performed in a dark state inside the laminar air flow with aseptic conditions. The medium used for growing the cells was discarded, and 2 mL of the prepared dye was added to the Petri plate and incubated for 2–3 min at 37 °C. Excess dye was washed using PBS, and later, the cell-loaded cover slip was placed on the frozen glass slide and visualized under a fluorescent microscope (Olympus BX51 fluorescent microscope with 10× magnification) with the live cells emitting a green color and the dead cells emitting a red color. Finally, the percentage of dead cells was determined by the following formula:

$$\%\mathrm{of}\mathrm{dead}\mathrm{cells}=\frac{\mathrm{Total}\mathrm{number}\mathrm{of}\mathrm{dead}\mathrm{cells}}{\mathrm{Total}\mathrm{number}\mathrm{of}\mathrm{cells}}\;\times \;100$$

### 3.14. Antibacterial Assay

The antibacterial assay was conducted to confirm the antibacterial effect of the synthesized hydrogels Alg/gel+Ag and Alg/gel+AgP following Bhuin et al. [50]. The study was performed against both Gram-positive (*Staphylococcus aureus*) and Gram-negative bacteria (*Escherichia coli*). The *E. coli and S. aureus* bacterial medium was sub-cultured in a fresh tube containing 7 mL Luria–Bertani (LB) broth and incubated overnight at 37 °C. Then, four cleaned, autoclaved glass test tubes were taken and labeled as blank (positive control), control (negative control), Alg/gel+Ag, and Alg/gel+AgP. The test tubes named blank contained 5 mL of LB broth, the control contained 4900 µL LB broth + 100 µL bacterial cultures, Alg/gel+Ag contained 3900 µL LB broth + 100 µL bacterial culture+ 1 mL of Alg/gel+Ag hydrogel, and Alg/gel+AgP contained 3900 µL LB broth + 100 µL bacterial culture+ 1 mL Alg/gel+AgP hydrogel, respectively. The tubes were then incubated in a shaking incubator for 24 h at 37 °C. Observation and photographs of the tubes were taken after 4 h and 24 h of incubation. Finally, the aliquots from the tubes (control, Alg/gel+Ag and Alg/gel+AgP) were plated on an agar plate and incubated for 24 h, and the number of colonies that formed was counted.

### 3.15. Minimum Inhibitory Concentration (MIC)

Following the antibacterial study, the MIC of both hydrogels was determined. The total volume of the mixture per tube was 5 mL. The hydrogels were serially diluted with the concentrations of 2.5 µg/mL, 4.5 µg/mL, 9.2 µg/mL, 18.4 µg/mL, 36.8 µg/mL, and 46 µg/mL, respectively; 100 µL of the bacterial cultures was used in this study. The tubes were incubated in a shaking incubator at 37 °C for 24 h. After 24 h, optical density (OD) was taken for the tubes to determine the MIC value for both the hydrogels Alg/gel+Ag and Alg/gel+AgP, respectively.

## 4. Conclusions

Wound healing is crucial for restoring skin and tissue function, involving several stages and activities. Both modern and traditional wound dressings aim to enhance healing, but each have their own set of limitations. Modern materials create an optimal environment but are not cost-effective, while traditional options are more affordable but may lack the necessary conditions for healing. To address this, nanomaterials have been explored as cost-effective alternatives that can provide the desired healing environment, offering various benefits due to their unique properties. In this study, we have prepared plant extracts like plantain peel, aloe vera, *Calendula officinalis*, and curcumin solution and utilized them for the healing of wounds along with silver nanoparticles. We have synthesized alginate/gelatin hydrogel both with nanosilver and with plant extracts and nanosilver. The hydrogel blended with the plant extracts provided various benefits due to the presence of bioactive compounds that offer many opportunities to create better wound healing than the hydrogel with nanosilver. The hydrogel Alg/gel+AgP provides a prominent effect in all aspects compared to the hydrogel Alg/gel+Ag. The Alg/gel+AgP had better stability and delivered 96% cell viability and 98% wound closure, even at the highest concentration of 5 µg/mL. Similarly, it exhibited efficient antibacterial activity for both *E. coli* and *S. aureus* bacteria with the MIC of 9.2 µg/mL and 18.4 µg/mL, respectively. These results prove that the synthesized hydrogel Alg/gel+AgP initiates enhanced wound healing with enhanced biocompatibility. This approach opens new possibilities for developing efficient wound healing products, which could address the problems related to the wound care management system. To address future directions for the developed hydrogels, it is essential to consider the initiation of clinical trials and additional in vivo studies to further validate their efficacy and safety. These studies are essential to thoroughly assess the hydrogels’ performance in real biological environments and to evaluate their safety and effectiveness for potential therapeutic applications. Conducting rigorous clinical trials will provide critical insights into dosage optimization, long-term effects, and patient-specific responses, ultimately facilitating the translation of these hydrogels from laboratory settings to clinical practice. Furthermore, in vivo studies can help to clarify the mechanisms of action and interactions with biological tissues, reinforcing the hydrogels’ potential as innovative wound-healing solutions.

## Data Availability

Data will be made available on request.

## Supplementary Materials

The following supporting information can be downloaded at https://www.mdpi.com/article/10.3390/molecules29215004/s1: Figure S1: Isolation of Plant extracts and synthesis of hydrogels title; Figure S2: Phytochemical and GC-MS analysis of isolated plant extracts: Table S1: Phytochemical constituents of Plant extracts title; Table S2: Phytochemical constituents of Plantain peel extract using GC-MS analysis; Table S3: Phytochemical constituents of Aloe vera extract using GC-MS analysis; Table S4: Phytochemical constituents of *Calendula officinalis* flower extract using GC-MS analysis; Table S5: Conformational bonds and functional group of the prepared plant extracts; Table S6: Conformational bonds and functional group of the synthesized hydrogels Alg/gel+Ag and Alg/gel+AgP. Table S7: The molecular formulae and the purity levels of all chemicals used in the study.
