# Supplementary material for: Enhancing Wound Healing with Nanohydrogel-Entrapped Plant Extracts and Nanosilver: An In Vitro Investigation
Source: Molecules. 2024 Oct 22;29(21):5004. doi: 10.3390/molecules29215004 (PMC11547294; doi:10.3390/molecules29215004)
Supplement: Supplementary file 1 [file molecules-29-05004-s001.zip › molecules-3255927-supplementary.pdf]

Table S1: Phytochemical composition of the isolated Plant extracts

S.No	Chemical test	Plantain peel extract	Aloe vera extract	*Calendula officinalis* flower extract
1	**Test for Saponins**	-	-	+++
2	**Test for Tannins** (Bayer's Test)	+++	+++	+++
3	**Test for Flavonoids** (Ferric chloride test)	-	-	-
4	**Test for Flavonoids** (Ferric chloride test)	-	+++	-
5	**Test for Alkaloids** (Mayer's test)	+++	+++	-
6	**Test for Glycosides**	-	-	-
7	**Test for Coumarins**	-	-	+++
8	**Test for Terpenoids and steroids** (Salkowski test)	-	-	+++
9	**Test for Quinones** (Sulphuric acid test)	-	-	+++
10	**Test for Carbohydrates** (Molisch's test)	+++	+++	+++
11	**Test for Proteins**	+++	-	-
12	**Presence of Reducing sugar** (Benedict's test)	+++	-	+++
13	**Presence of Polysaccharide**	-	-	-

Table S2: Phytochemical constituents of Plantain peel extract using GC-MS analysis

S No	Chemical constituent	Molecular weight	Formula	R.T (min)	Peak area percentage (%)
1	Betulin	442	$C_{30}H_{50}O_2$	41.397	67.75
2	2-(4-{[4-oxo-3-phenyl-2-(phenylimino)-1,3-thiazolidin-5-ylidene]methyl}phenoxy)acetamide 2-[4-(4-oxo-3-phenyl-2-phenylimino-thiazolidin-5-ylidenemethyl)-phenoxy]-acetamide	429	$C_{24}H_{19}N_3O_3S$	42.395	3.65

3	2-(€-[(€-2-([€-(2-hydroxyphenyl)methylidene]amino)propyl)imino]methyl)phenol.alpha.,.alpha.’-(1-methylethylenediimino)di-ortho-cresol .alpha.,.alpha.’-dipropylenedinitrilodi-o-cresol 2,2’-[(1-methyl-1,2-ethanediyl)bis(nitrilometh)	282	$C_{17}H_{18}N_2O_2$	42.484	12.92
4	Bicyclo[4.3.0]nonane, 4,5-dimethyl-1-(1-hydroxy-2-propyl)-5-(3-methyl-2-pentenoyl)- (2Z)-1-[7a-(2-Hydroxy-1-methylethyl)-4,5-dimethyloctahydro-1H-inden-4-yl]-3-methyl-2-penten-1-one	306	$C_{20}H_{34}O_2$	44.465	7.15
5	1,8-Dioxa-5-thiaoctane, 8-(9-borabicyclo[3.3.1]non-9-yl)-3-(9-borabicyclo[3.3.1]non-9-yloxy)-1-phenyl- 2-(9-Borabicyclo[3.3.1]non-9-yloxy)-3-([2-(9-borabicyclo[3.3.1]non-9-yloxy)ethyl]sulfanyl)propyl phenyl ether	468	$C_{27}H_{42}B_2O_3S$	44.634	8.52

Table S3: Phytochemical constituents of Aloe vera extract using GC-MS analysis

S No	Chemical constituent	Molecular weight	Formula	R.T (min)	Peak area percentage (%)
1	Diethyl Phthalate 1,2-Benzenedicarboxylic acid, diethyl ester Phthalic acid, diethyl ester o-Benzenedicarboxylic acid, diethyl ester Anozol Ethyl phthalate Neantine Palatinol A Phthalol Placidol E Solvanol Unimoll	222	$C_{12}H_{14}O_4$	16.860	64.62
2	1,2-Benzenedicarboxylic acid, diethyl ester phthalic acid - benzenedicarboxylic acid diethyl ester 1, 2-benzenedicarboxylic acid, diethyl ester 1,2-benzenedicarboxylic acid, diethyl ester	222	$C_{12}H_{14}O4$	17.361	12.22
3	Heneicosane n-Heneicosane	296	$C_{21}H_{44}$	18.074	4.06

	Henicosane				
4	Diethyl Phthalate 1,2-Benzenedicarboxylic acid, diethyl ester Phthalic acid, diethyl ester o-Benzenedicarboxylic acid, diethyl ester Anozol Ethyl phthalate Neantine Palatinol A Phthalol Placidol E Solvanol Unimoll	222	$C_{12}H_{14}O_4$	18.258	16.99
5	2',3'-Dihydro-2',3'-epoxysatratoxin h brn 4623147 satratoxin h, 2',3'-dihydro-2',3'-epoxy-spiro(10,12:19,22a-dimethano-4h,5h,22ah-oxireno(8,9)(1,6,12)trioxacyclooctadecino(3,4-d)(1)benzopyran-11(10h),2'-oxirane)-2,14(1ah,	544	$C_{28}H_{32}O_{11}$	44.614	2.10

Table S4: Phytochemical constituents of *Calendula officinalis* flower extract using GC-MS analysis

S No	Chemical constituent	Molecular weight	Formula	R.T (min)	Peak area percentage (%)
1	Anhydro-1,6-dideoxyhexo-3,4-diulose 2,5-dimethyl-3(2h)furanone 2,5-dimethyl-3-hydroxy-4-oxo-4,5-dihydrofuran 2,5-dimethyl-4-hydroxy- 3(2h)-furanone (dmhf) 2,5-dimethyl-4-hydroxy-2,3-dihydrofuran-3-one 2,5-dimethyl-4-hydroxy-3(2h)	107.58	C6H8O3	3.666	13.66
2	Propanal, 2,3-dihydroxy-, (S)- 2,3-Dihydroxypropanal L-Glyceraldehyde	398.7	C3H6O3	4.900	12.81
3	4H-Pyran-4-one, 2,3-dihydro-3,5-dihydroxy-6-methyl- 3,5-Dihydroxy-6-methyl-2,3-dihydro-4H-pyran-4-one 2,3-dihydro-3,5-dihydroxy—6-methyl-4H-pyran-4-one Pyranone 2,3-Dihydro-3,5-dihydroxy-6-methyl-4-pyrone 3-Hydroxy-2,3-dihydromalto	518.52	C6H8O4	5.149	22.28
4	Diethyl Phthalate 1,2-Benzenedicarboxylic acid, diethyl ester Phthalic acid, diethyl ester o-	116.16	C12H14O4	18.255	22.95

	Benzenedicarboxylic acid, diethyl ester Anozol Ethyl phthalate Neantine Palatinol A Phthalol Placidol E Solvanol Unimoll				
5	2(4h)-benzofuranone, 5,6,7,7a-tetrahydro-6-hydroxy-4,4,7a-trimethyl-, (6s-cis)- (-)-loliolide (-)loliolide 1,3-dihydroxy-3,5,5-trimethylcyclohexylidene-4-acetic acid lactone 2(4h)-benzofuranone, 5,6,7,7a-tetrahydro-6.beta.-hydroxy-4,4,	185.31	C11H16O3	24.427	28.30

Table S5: Conformational bonds and functional group of the prepared plant extracts

S.No	Wavenumber	Functional bonds	Functional groups
**Plantain peel**			
1	3425	O-H stretch, H-bonded	Alcohols, phenols
2	2923	C-H stretch	Aromatics
3	2848	H-C=O: C-H stretch	Aldehydes
4	1633	C-H bend	Alkanes
5	1459	C-N stretch	Aliphatic amines
**Aloe vera**			
1	3445	O-H stretch, H-bonded	Alcohols, phenols
2	2913	C-H stretch	Aromatics
3	2852	C-H stretch	Alkanes
4	1728	-C=H bend	Aldehydes, saturated aliphatic
5	1641	N-O symmetric stretch	Nitro compounds
6	1040	C-N stretch	Aliphatic amines
***Calendula officinalis* flower**			
1	3445	O-H stretch, H-	Alcohols, phenols

		bonded	
2	2913	C-H stretch	Aromatics
3	2852	C-H stretch	Alkanes
4	1728	-C=H bend	Aldehydes, saturated aliphatic
5	1641	N-O symmetric stretch	Nitro compounds
6	1040	C-N stretch	Aliphatic amines
**Curcumin**			
1	3514	O-H stretch	Free hydroxyl
2	2915	C-H stretch	Alkanes
3	2845	C-H stretch	Alkanes
4	1507	C-C stretch (in ring)	Aromatics
5	1281	C-H wag (-CH_2X)	Alkyl halides

Table S6: Conformational bonds and functional group of the synthesized hydrogels Alg/gel+Ag and Alg/gel+AgP

S.No	Wavenumber	Functional bonds	Functional groups
**Alg/gel+Ag**			
1	3316	O-H stretch, H-bonded	Alcohols, phenols
2	1636	C-C stretch	Alkenes
3	1308	C-C stretch	Alcohols, carboxylic acids, esters
**Alg/gel+AgP**			
1	3403	O-H stretch, H-bonded	Alcohols, phenols
2	1642	C=C stretch	Alkenes
3	1330	N-O symmetric stretch	Nitro compounds

Table S7: The molecular formulae and the purity levels of all chemicals used in the study

S.No	Chemicals used	Molecular formulae	Purity levels
1	Sodium alginate	$C_6H_8O_6$	≥ 90% (depending on the grade used for research or food purposes)
2	Gelatin	-	98–99% (usually denoted by bloom strength for gel-forming capacity)
3	Silver Nitrate	$AgNO_3$	≥ 99.9% (analytical reagent grade)
4	Acetone	C_3H_6O	99.5% or higher (analytical grade)
5	Glutaraldehyde	$C_5H_8O_3$	25%–50% (usually supplied in aqueous solution)
6	Calcium chloride	$CaCl_2$	95–99% (lab-grade and food-grade can vary)

Figure S1 Isolation of Plant extracts and synthesis of hydrogels

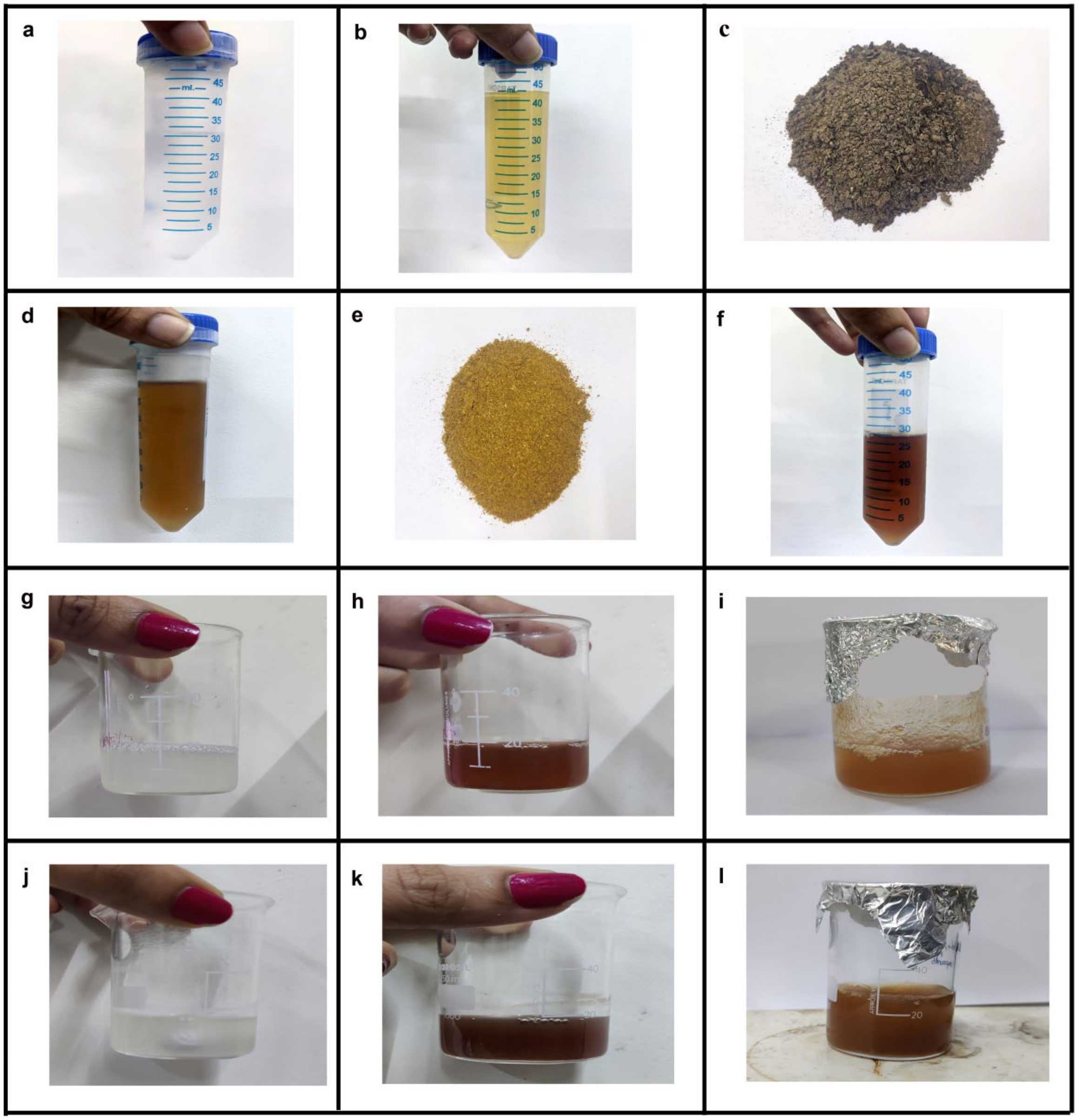

Fig.S1. **(a)** Prepared Aloe vera extract **(b)** Prepared Curcumin solution **(c)** Shade dried plantain peel powder **(d)** Prepared Plantain peel extract **(e)** Commercial *Calendula officinalis* flower powder **(f)** Prepared *Calendula officinalis* flower extract **(g)** The hydrogel Alg/gel+Ag before UV irradiation **(h)** The hydrogel Alg/gel+Ag after UV irradiation **(i)** Final Alg/gel+Ag hydrogel **(j)** The hydrogel Alg/gel+AgP before UV irradiation **(k)** The hydrogel Alg/gel+AgP after UV irradiation **(l)** Final Alg/gel+AgP hydrogel.

Figure S2 Phytochemical analysis of the isolated plant extracts

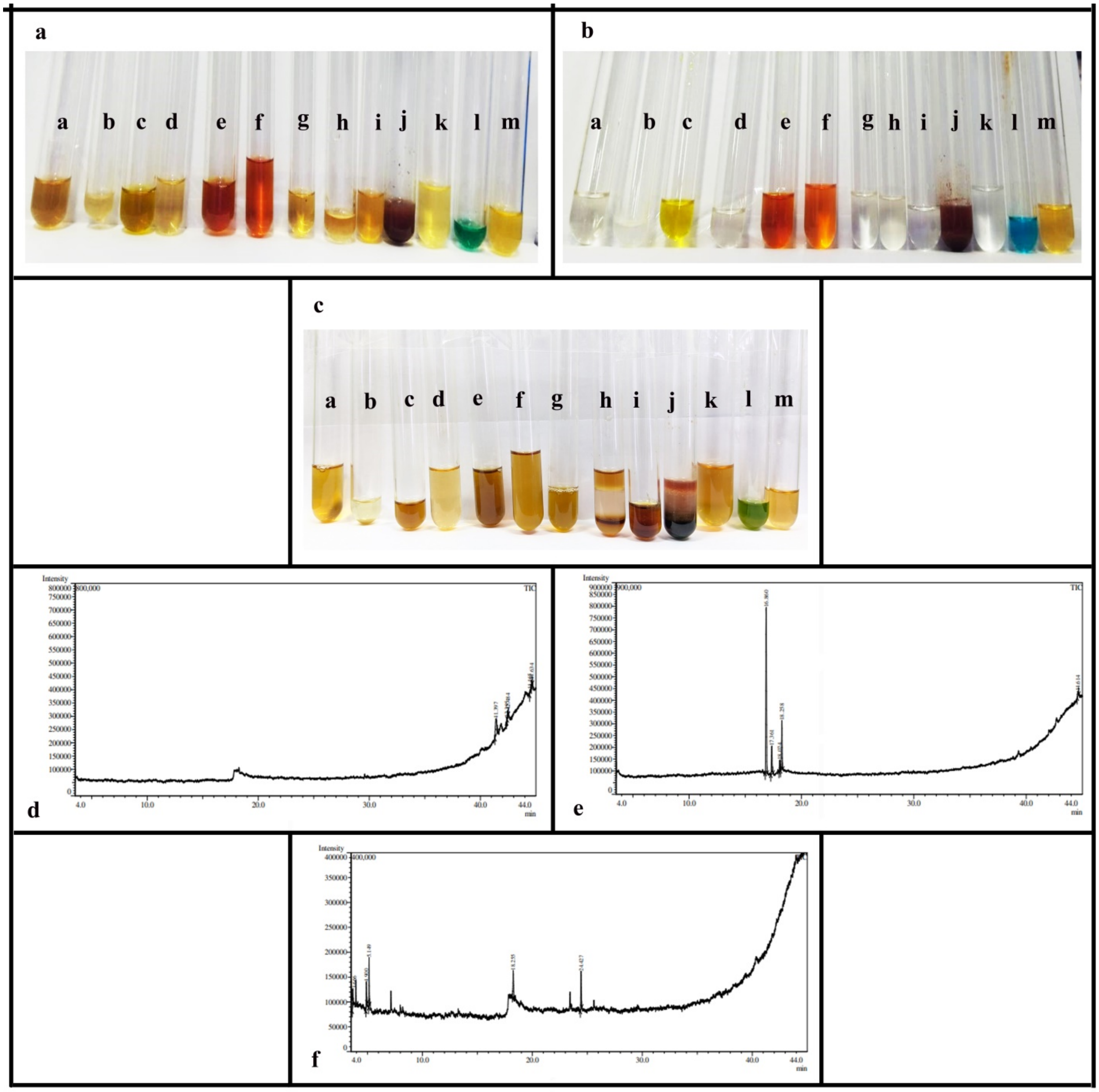

Fig.S2. Phytochemical analysis of **(a)** Prepared Plantain peel extract **(b)** Prepared Aloe vera extract **(c)** Prepared *Calendula officinalis* flower extract. GC-MS analysis of **(d)** Prepared Plantain peel extract **(e)** Prepared Aloe vera extract **(f)** Prepared *Calendula officinalis* flower extract
